# Realizing the Potential of Adolescence to Prevent Transgenerational Conditioning of Noncommunicable Disease Risk: Multi-Sectoral Design Frameworks

**DOI:** 10.3390/healthcare4030039

**Published:** 2016-07-04

**Authors:** Jacquie L. Bay, Susan M. Morton, Mark H. Vickers

**Affiliations:** 1 Liggins Institute, University of Auckland, Auckland 1142, New Zealand; s.morton@auckland.ac.nz (S.M.M.); m.vickers@auckland.ac.nz (M.H.V.); 2 Centre for Longitudinal Research-He Ara ki Mua, University of Auckland, Auckland 1743, New Zealand

**Keywords:** adolescence, noncommunicable disease risk, obesity, school-based, knowledge translation, developmental origins of health and disease (DOHaD), multi-sectoral, complexity

## Abstract

Evidence from the field of Developmental Origins of Health and Disease (DOHaD) demonstrates that early life environmental exposures impact later-life risk of non-communicable diseases (NCDs). This has revealed the transgenerational nature of NCD risk, thus demonstrating that interventions to improve environmental exposures during early life offer important potential for primary prevention of DOHaD-related NCDs. Based on this evidence, the prospect of multi-sectoral approaches to enable primary NCD risk reduction has been highlighted in major international reports. It is agreed that pregnancy, lactation and early childhood offer significant intervention opportunities. However, the importance of interventions that establish positive behaviors impacting nutritional and non-nutritional environmental exposures in the pre-conceptual period in both males and females, thus capturing the full potential of DOHaD, must not be overlooked. Adolescence, a period where life-long health-related behaviors are established, is therefore an important life-stage for DOHaD-informed intervention. DOHaD evidence underpinning this potential is well documented. However, there is a gap in the literature with respect to combined application of theoretical evidence from science, education and public health to inform intervention design. This paper addresses this gap, presenting a review of evidence informing theoretical frameworks for adolescent DOHaD interventions that is accessible collectively to all relevant sectors.

## 1. Introduction

Escalating rates of obesity and associated avoidable noncommunicable diseases (NCDs) have increased global attention on impacts, prevention, and control. NCDs, primarily cardiovascular diseases, cancers, chronic respiratory diseases and type 2 diabetes mellitus, are responsible for 68% of global deaths, with 40% occurring prematurely (before age 70). Low- and middle-income nations bear 75% of all, and 82% of premature, NCD deaths [1]. The World Health Organization (WHO) and United Nations (UN) have heightened societal awareness of this issue, calling for multi-sectoral evidence-based actions focused on prevention and equity [2].

Principles and approaches guiding fulfillment of the vision, “*a world free of the avoidable burden of NCDs*”, propose multi-sectoral, empowerment-based knowledge-translation strategies, including life-course approaches to prevention and control [3]. Life-course approaches draw on evidence from the field of developmental origins of health and disease (DOHaD). This identifies that nutritional and non-nutritional exposures during development (from gametogenesis to early childhood) contribute towards later-life NCD vulnerability [4], and that maternal health (including preconception), and maternal, infant, childhood and adolescent nutritional quality, contribute to NCD prevention and control [3].

Pregnancy, lactation and infancy were rapidly identified as life-stages appropriate for DOHaD-informed interventions [5,6]. However, evidence demonstrates that health prior to conception, along with nutritional environments during the periconceptional period, contribute to programming/conditioning of later-life health and disease [4]. Therefore, addressing determinants of health prior to conception, and periconceptional environmental exposures, is extremely important.

Adolescence is a determining point for nutritional, physical activity, and cognitive behaviors that persist into adulthood [7,8] and influence future health [9]. Consequently, these behaviors will influence periconceptional environmental exposures and health prior to conception. Even if pregnancy is a considerable distance from adolescence, behaviors that develop during adolescence contribute towards later-life NCD vulnerability in offspring. Thus, adolescence is a life-stage offering significant potential for transgenerational primary prevention of obesity and NCD risk [10].

Reports recommending inclusion of adolescent interventions within prevention strategies suggest multi-sectoral approaches, highlighting the potential of schools as intervention settings [3,11,12]. Realizing this requires education to partner with health and science in intervention design, delivery, and evaluation. While multiple other intervention foci are possible (including community groups-, family-, and healthcare-based approaches), our emphasis is on the potential identified by school-based interventions and the theoretical frameworks informing these.

Having led the development of multi-sectoral education–science–health adolescent intervention partnerships in Oceania [13,14], and supported UK colleagues to develop culturally adapted versions of our interventions [15], we observe that even when key multi-sectoral partnership success factors of shared vision, strong relationships, and resourcing [16] are available, it is challenging for partners to understand and integrate contributing perspectives into intervention design. We suggest that sector-specific literature exacerbates this challenge. This paper, intended for science, health and education audiences, addresses a gap in the literature, examining conceptual frameworks underpinning adolescent DOHaD intervention design from a *multi-sectoral perspective*. It is intended as an enabling tool supporting participating sectors to recognize sectoral-specific perspectives. From this collaborators may gain understanding of evidence and drivers from partner sectors, and consider how evidence and practice can be integrated to achieve the potential of transgenerational NCD risk reduction offered within adolescence.

## 2. Multi-Sectoral Partnerships

Multi-sectoral partnerships are recommended where complexity determines that no single sector has the required expertise or resources to bring about change [17]. It has been identified that this is the case with the NCD epidemic [3,11]. Multi-sectoral partnerships are known to be challenging, time consuming, and often fail. However, when multi-sectoral partnerships are successful, they achieve more collectively than partners achieve alone [18]. When addressing the potential for DOHaD-informed interventions supporting NCD risk reduction for adolescents and their potential future offspring, key partners are education, science, health, and social/community agencies.

### 2.1. Schools: A Setting for Multi-Sectoral Adolescent Intervention

Multiple social structures offer settings for DOHaD-driven interventions aiming to empower adolescents to engage in evidence-based preventive actions. Settings need to enable individuals and/or communities to examine evidence (health, scientific and sociological), identify the significance of this evidence for their context, and identify and engage in evidence-based risk-reducing actions. To facilitate the potential of positive health-behaviors prior to parenting, intervention settings should engage adolescents before and within the reproductive years. Participation should be sustained for long enough to allow in-depth interaction and effect learning and development that leads to evidence-based actions. Intervention settings sit within and are impacted by the social, environmental and political context of the community. This influences resourcing (available and required) to support adolescent empowerment, the type of actions adolescents will identify as opportunities for change, and the manner in which adolescents may approach empowerment-based social disruption to bring about change in the health status of their families and communities.

Schools offer established social networks wherein education can facilitate development of competencies (knowledge, attitudes, skills and values) that empower adolescent-led evidence-based actions within the socio-ecological context of the individual and their community [13]. However, not all adolescents are positively connected with schooling, therefore community-based interventions are also important. Cultural, service, religious, sporting, or hobby-based groups that meet regularly and engage in informal or formal learning linked to themes associated with health and wellbeing, offer further intervention settings for adolescents and adults [19,20].

### 2.2. Shared Vision within Multi-Sectoral Partnerships

Shared vision is the most significant critical success factor for multi-sectoral partnerships [18]. Also required are: resources (human and other), leadership, organizational structure and capacity, appropriate membership, quality of relationships, and understanding of external and contextual factors [16]. This list could be applied equally to multi-disciplinary collaborations. However, unlike multi-disciplinary interactions, multi-sectoral partnerships require negotiation of language, knowledge, and practice across sectors. Furthermore, sector-specific goals essential to achieving shared goals must be recognized and respected.

Within multi-sectoral partnerships it is known that differing goals lead to devaluing of others’ strategies by partners, and that this limits success [21]. In school-based health-promoting programs it is reported that lack of connection between the program and the core mission of the school is the most common reason for failure [22]. This suggests that program goals are typically not shared by the participating sectors, and lack relevance to education. Addressing this issue is essential.

**Figure 1:**
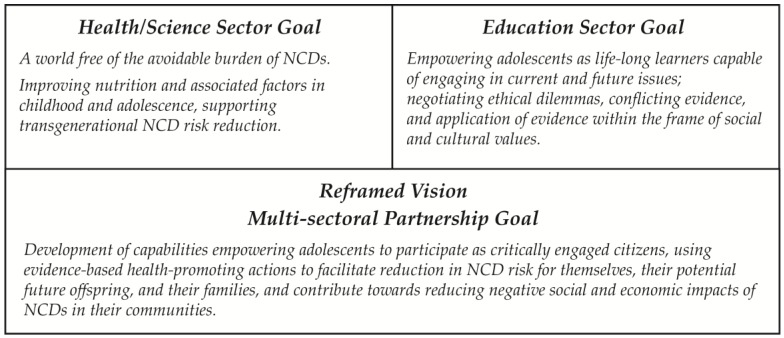
Reframing of sector-specific goals to enable a multi-sectoral goal that identifies shared vision while including and respecting sector-specific vision.

The science and health sectors share goals related to improving nutrition and associated factors in childhood and adolescence, promoting transgenerational NCD risk reduction [3,11,12]. While health and wellbeing are important to the education sector, overarching goals in education focus on development of competencies empowering adolescents as life-long learners capable of engaging in current and future issues; negotiating ethical dilemmas, conflicting evidence, and application of evidence within the frame of social and cultural values [23]. Although these sector-specific goals for education are different to those for health and science, when integrated the combined goals offer the potential to enhance outcomes for each sector. By reframing the health/science vision relating to transgenerational NCD risk reduction to include related educational goals, it is possible to create a vision that all participating sectors can value, Figure 1. This mandates recognition of each sector in planning, development, delivery, and evaluation.

Achieving shared vision requires investment in partnership development, including recognition and respect for sector-specific goals [14]. The impact of investment in inter-sectoral consultation was demonstrated recently in the published reports (from draft to final) of the WHO Commission on Ending Childhood Obesity (ECHO) [12,24,25]. The final report proposes school-based interventions as one component of the six strategies identified to support reduction in childhood obesity and contribute towards long-term NCD risk reduction. It identifies the need for engagement and consultation with education, acknowledging that interventions must have educational rigor, be co-constructed with teachers and embedded within mainstream curricula [12]. Inclusion of this statement, not seen in the interim report [24], followed consultation with education [26]. This transactional process supported recognition and respect of partner sector perspectives. By investing in inter-sectoral consultation the Commission moved the report from a draft document imposing health-driven guidelines on education, to a document proposing action via multi-sectoral partnership. This significant change in approach offers potential to address recurrent failure of school-based health interventions resultant from lack of connection with the core mission of schools [22].

### 2.3. Partnership Engagement

Education, often seen as a panacea for multiple social ills, may react skeptically when yet another issue to be addressed in schools is identified. While science/health has recognized adolescence as a valid point to disrupt transgenerational obesity/NCD cycles, this is not core business for education. To accept and sustain the invitation to lead intervention design and facilitation, the education sector (both leaders and teachers) needs to examine the relevant scientific and health evidence in depth, and consider the potential for the NCD/DOHaD context to support educational goals. We intentionally define education as the leader of intervention design. Health and education leaders within our DOHaD translation partnership programs believe that inviting education to lead intervention design has achieved significantly more than when health develop and offer interventions to schools [27], which inevitably do not fit with schools’ core missions, and as commonly cited [22], as a result are not sustained.

Non-educators involved in partnerships need to appreciate modern pedagogy and practice. Shifts in understanding of the nature of knowledge (epistemology) in the past 20 years have seen a move from positivism towards constructivism, promoting learner-centered classrooms [28]. (Positivism refers to a position where knowledge is only determined by empirical observation. Constructivism identifies that people construct understanding of new knowledge based on previous knowledge, understanding and experiences.) While this move towards learner-centered classrooms promotes development of competencies recognized as essential for 21st century learners [23], it also challenges the enduring belief that teaching is *“primarily about the transmission of subject matter from teacher to student*” [29] (paragraph 11). Multi-sectoral engagement must therefore provide opportunities for partners to explore and challenge personal attitudes, beliefs and perceptions (about NCDs and education), enabling others’ perspectives to be considered.

## 3. Intervention Design: Theoretical Underpinnings

It is appropriate that intervention design is informed by the core contributing fields; science, education, public health. This adds challenges to the process of intervention design as each contributing sector must engage with and develop an appreciation of all relevant evidence. We present in this section important theoretical contributions from relevant contributing sectors.

### 3.1. Sense-Making in Complex Adaptive Systems: Challenging Reductionist Thinking

Intervention design facilitating multi-sectoral action to empower adolescents as agents of transgenerational NCD risk reduction can be usefully informed by complexity theory, *“a theory of change, evolution, adaptation and development for survival”*, which challenges reductionist approaches to understanding phenomena [30]. Both the NCD epidemic [31] and schools [30] are classified as complex adaptive systems. They are dynamic, involving multiple elements interacting non-linearly with each other and the environment. Elements evolve in response to interactions, influenced by historical and current settings. Small changes within the system may produce disproportionately significant consequences [32,33]. Thus, actions to address transgenerational NCD risk require change about and within interacting complex adaptive systems.

Sense-making is a social process involving exploration and interpretation of divergent perspectives to make sense of and respond to current and future issues [34]. The Cynefin model, describing knowledge in relation to cause and effect as known, knowable, complex (or emergent) and chaotic [35] supports this process. While developed for organizational management, it is applicable to health [36] and education [37], and useful within multi-sectoral planning and resultant interventions.

Reductionist thinking, promoting individual responsibility or blame over understanding of complex social and biological determinants of risk, is frequently used in relation to NCDs/DOHaD, particularly at the level of public communication. Legitimate in the context of defined best-practice, where cause and effect relationships are, or can be, made evident to everyone, this perspective is inadequate in relation to causality of NCD risk. Application of the Cynefin sense-making approach to inform health promotion strategies addressing complex issues *“challenges preferential engagement with ‘down-stream’ issues and validates contextualized emergent practice within communities when working with complex issues”* [36]. In relation to school-based interventions, sense-making, because it focuses on exploring multiple perspectives, can support:

- intervention design by multi-sectoral teams;
- contextual adaptation and integration of interventions within schools; and
- age-appropriate exploration of dynamic relationships between knowledge (known, knowable and emergent) and system agents contributing to the determination of NCD risk by participating adolescents, leading to evidence-based decision-making.

### 3.2. DOHaD: Biological Evidence Supporting Adolescent Intervention

The rationale for primary and transgenerational prevention of NCD risk via adolescent intervention, outlined briefly in the introduction, emerges from the field of DOHaD. This evidence must be made accessible to all partners in a manner that enables it to be understood and contextually interpreted by program facilitators.

In animal models, under-nutrition and obesogenic early life environments result in offspring with a propensity towards subsequent development of metabolic or cardiovascular diseases [38]. Evidence exploring the timing of environmental insults impacting development shows that from gametogenesis through fetal development, impacts from environmental exposures may *contribute towards* adverse long-term outcomes [39,40]. Longitudinal human cohort studies have identified the contribution of early life environmental exposures to long-term health and wellbeing within differing socio-ecological settings [41,42,43]. Importantly, these data highlight relationships between sociological and physiological determinants of long-term health. We emphasize contribution, rather than absolute determination of adverse outcomes, a point important in communicating and translating DOHaD evidence.

Evidence of mechanisms underpinning the impact of early life environmental exposures on later-life health has demonstrated that during critical life-course periods, developmental plasticity creates the potential for environmental exposures to influence regulatory pathways by altering gene expression in response to favorable or unfavorable conditions. The latent phenotypic influences resulting from developmental plasticity may be associated with increased neuro-cognitive developmental challenges [44,45], early puberty [46], overweight and obesity, metabolic syndrome [47,48], type 2 diabetes mellitus, cardiovascular diseases [49], osteoporosis [50], allergic diseases [51], affective disorders [44,52], and altered reproductive function [53]. Current evidence strongly supports the hypothesis that epigenetic mechanisms, such as DNA methylation, histone acetylation and non-coding RNA expression, are responsible, at least in part, for altered phenotypes [42,54,55]. In some cases these changes could be transmitted across generations as well as between mother and fetus [56,57], and are not always stable, offering the potential for modification via targeted nutritional or other intervention [58].

**Figure 2:**
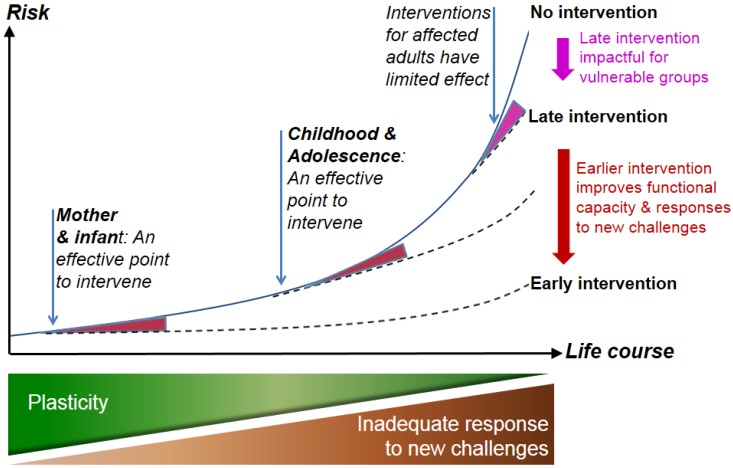
Life-course view of noncommunicable disease (NCD) risk. Risk increases in a nonlinear way as a result of declining plasticity and accumulative damage from lifestyle-imposed or other challenges. The effect of mismatch between developmentally and evolutionarily influenced phenotype and adult environment also increases through the life-course. Interventions in adults, especially those at high risk, can be beneficial, but only to a degree. Screening in middle-aged adults may also be too late to reduce risk substantially. Interventions in adolescents and young adults are likely to be more effective and, importantly, can reduce the risk of NCDs in the next generation. The prenatal period establishes risk through interaction between genetic, epigenetic and environmental factors. (From Hanson and Gluckman 2014, *Physiology Review* 94: 1027–1076, with permission.)

Collectively, these data suggest that two forms of preventive intervention are theoretically possible. First, by improving adverse nutritional and environmental exposures before and during pregnancy, predictive adaptive or conditional responses that lead to phenotypic vulnerabilities such as increased later-life NCD risk could be minimized, Figure 2. Secondly, the potential to identify mechanisms to reverse epigenetically mediated modulation of gene expression for key genes and regulatory pathways could lead to focused interventions. Currently, very limited evidence of the potential to reverse the impacts of programming exists and is predominantly based on outcomes from experimental animal models [38,59]; therefore significant further evidence is required before such approaches are considered. This leaves behavior change relevant to improving environmental exposures prior to and during pregnancy, childhood and adolescence as the primary focus for interventions. Combined with evidence of the role of adolescence in the setting of health-related behaviors (described earlier), this established the biological basis for DOHaD interventions during adolescence. To design and facilitate school-based interventions, teachers, no matter what their discipline, should be provided with opportunities to examine and make sense of these data.

### 3.3. Science Communication: Challenging Transmission, Promoting Transaction

DOHaD research evidence has reached a point where, if applied in community settings, it has the potential to improve health and wellbeing (social and economic). This does not mean that new evidence will not continue to emerge, but does mean that the right of every human to “*share in scientific advancement and its benefits”* [60] must be respected. Thus, effective communication and translation of DOHaD research evidence must be prioritized.

Core to this science communication/translation process is the concept that programs must facilitate contextual interpretation of evidence within communities for whom it has relevance, so that individuals and/or groups can decide how to use (or not use) the evidence. Hence, we need to examine the concept of science communication, and how this is interpreted by sectoral partners within intervention design to enable individuals and communities (not scientists and health professionals) to decide on contextually relevant evidence-based actions.

Traditional views of science communication, defined by positivist epistemology, identify knowledge as descriptions of phenomena that can be observed, measured and absolute. This leads to the belief that knowledge can be transmitted from expert to public, creating an informed society that uses scientific evidence in decision-making [61]. Established by the 1980s, Public Understanding of Science (PUS) movement and underpinned by deficit models of learning [61,62], transmission-based communication models lack meaningful engagement, supporting neither development of trust nor empowerment of citizens to use science knowledge in decision-making [63]. More enlightened ideals associated with the Science in Society movement [62], alongside concepts of Knowledge Translation, Knowledge Exchange, and Integrated Knowledge Translation [64], support transactional communication and engagement. These recognize that knowledge application is situated, and that requirements for interaction between research and knowledge-user communities necessitate this process to be social [65], and consequently dependent on relationships. This represents a constructivist epistemology, common in education. Constructivism identifies that because knowledge is situated, it cannot be transmitted, meaning that individuals construct understanding based on previous knowledge and experiences [66]. However, what do we mean by knowledge? Should we be discussing knowledge at all? Has it not been proven that knowledge does not contribute to behavior change? While it is known that knowledge does not necessarily lead to behavior change [67], it is also known that knowledge is necessary, but not the sole factor required to facilitate behavior change [68].

Knowledge is a multi-dimensional concept that encompasses more than a collection of facts. It can be categorized as declarative (or content), procedural, and epistemic. The ability to use knowledge in decision-making is influenced by attitudes, competencies and context. In modern schooling, rather than transmitting declarative knowledge, teachers facilitate learning experiences that allow students to examine, question, challenge, and formulate their own ideas, opinions, and conclusions [66]. As well as supporting the construction of understanding such learning promotes: exploration of values and dispositions; development of skills associated with interpretation and analysis of evidence from varying perspectives; and capabilities associated with acting on evidence. This process is learner-centered and transactional, recognizing that social processes support students to question beliefs and negotiate uncertainty and diverse ideas. This does not reject core disciplinary knowledge. Teachers facilitate opportunities for students to access established disciplinary knowledge from which they can examine their own and others’ perspectives [23].

When research evidence is explored in open, non-linear learning environments that present multiple perspectives on an issue alongside core scientific and sociological concepts, development of understanding that leads to conscious decision-making initiated by adolescents is possible [13]. This transactional process is enabled via use of narrative, known to facilitate learning and enable access to evidence [69,70]. Therefore, teachers need access to narratives that humanize the process by which this evidence has evolved [71,72,73], enabling exploration of the journey of scientific and/or sociological discovery, as well as stories of NCDs and their impacts within communities [74]. These narratives simultaneously facilitate development the capabilities required to negotiate evidence and complex socio-scientific issues.

Transactional, learner-centered programs that facilitate construction of meaningful understanding of evidence from which decision-making can emerge differ significantly from transmission-based science communication, such as that used to facilitate mass public awareness. Campaigns such as “5+ a day” transmit declarative knowledge via mass media, pamphlets, posters etc. without the opportunity to enable the public to engage with evidence that has led to the knowledge. Furthermore in such programs little opportunity exists for collaborative construction of contextual understanding of this knowledge. Thus, it is not surprising that such programs are known to improve awareness but not change behaviors [75]. While often labeled as “education programs”, use of the term education in this context differs significantly from education as a transactional learner-centered process. Therefore it is important to unpack what we mean by education, as when working across multi-sectoral science-health-education partnerships we have identified that differing understanding of terms such as education, curriculum, knowledge, capabilities and learning are a key issue that if not addressed can lead to significant misunderstanding.

### 3.4. Complexity of Risk and Impact: Why Educators are so Enthusiastic about NCDs

The complexity of NCD cause and effect must underpin intervention design. This complexity, while challenging, presents significant educational opportunity with respect to development of competencies enabling critical engagement in open-ended social issues [23], validating partnership engagement for education. From a citizen’s perspective, the challenges in making sense of NCD cause and effect stem from the:

- extremely broad profiles of NCDs as a disease cluster;
- latency between environmental exposure and potential identification of risk;
- extended time over which risk and morbidity develops;
- breadth of physiological systems that may be impacted; and
- complex interaction of sociological, environmental, genetic and epigenetic factors that contribute to NCD risk profiles for individuals, families, and populations, including
  ○ the double burden of maternal and child malnutrition alongside child and/or adult overweight, obesity and NCDs found in low- and middle-income countries and socially/economically disadvantaged populations, and
  ○ impacts emerging from climate change, nutritional transitions, and food insecurity.

DOHaD evidence is a component of this complexity. The impact of early life exposures on later-life NCD risk is neither isolated nor absolute, but resides within a life-course approach to understanding NCD risk, disease and prevention [76]. Furthermore, DOHaD mechanistic evidence frequently comes from tightly controlled animal models. When over-simplified, these models can inadvertently suggest individual blame, with the mother’s actions determining outcomes in the next generation [77]. While this contrasts from commonly held views of individual lifestyle determination of NCD risk, both are inadequate.

This matrix of interacting factors impacting NCD risk creates multiple rich contexts for learning that is designed to facilitate development of capabilities required for critical engaged citizenship. Intervention design should enable age-appropriate exploration of current and historical influences on and beliefs about NCD risk, facilitating students to identify and challenge beliefs and assumptions (personal, family, and societal) and negotiate concepts of: association rather than determination; transgenerational risk; and interaction between environmental and biological factors within a broader societal context. Learning such as this supports competency development, and empowers evidence-based actions.

### 3.5. Capabilities Required to Negotiate Socio-Scientific Issues: Partnership Value of and for Education

Initiated by the 1970s Science, Technology and Society movement, examination of socio-scientific issues is a universally agreed component of schooling [78,79]. Despite extensive literature examining learning frameworks [80,81], science education remained strongly focused on development of universal and decontextualized knowledge [82], lacking exploration of social and political interactions with science. Only recently has education planning and design examined the need for adolescents to develop capabilities required to engage actively with open-ended, complex, and socially relevant issues [23] within and across multiple subject areas. These capabilities encompass key competencies, scientific and health literacies, and self-efficacy. Their development via exploration of NCDs should be central to intervention design and offers validity to the education sector in joining partnerships.

#### 3.5.1. Key Competencies

In response to challenges of increasing social complexity, the education sector, via the Development and Selection of Competencies (DeSeCo) program, defined competencies required for individuals (beyond the basics of reading, writing, and calculating) to “*lead an overall successful and responsible life and for contemporary society to face present and future challenges*” [83]. They sit within a matrix of context-specific capabilities associated with:

- interactive use of tools (language, text, knowledge, information, technologies);
- interactions within heterogeneous groups (the ability to relate well to others, cooperate, manage and resolve conflict); and
- the ability to act autonomously (act within the big picture, form and conduct life-plans and personal projects; defend and assert rights, interests, limits, and needs) [84].

In the context of NCDs, these competencies contribute to the ability of adolescents to engage with evidence (sociological and scientific) and decide how individual, community and societal actions may be beneficial. Capabilities associated with knowledge and understanding of science and health are also required.

#### 3.5.2. Health Literacy

Health literacy describes a set of capabilities supporting and enabling evidence-based decision-making related to wellbeing and health at personal, community and societal levels and is therefore an important aspect of intervention design. While definitions are variable and at times confined to health-service literacy, the asset model of health literacy proposed by Nutbeam considers the capabilities required to enable critical informed engagement in decision-making. “*Health literacy implies the achievement of a level of knowledge, personal skills and confidence to take action to improve personal and community health by changing personal lifestyles and living conditions. Thus, health literacy means more than being able to read pamphlets and make appointments. By improving people’s access to health information, and their capacity to use it effectively, health literacy is critical to empowerment.*” [85].

Nutbeam’s ongoing refinement of this model presents the capabilities within a frame of increasing cognitive demand. Useful from an educational perspective, it moves from basic/functional literacy, associated with skills for daily living, towards communicative/interactive literacy, and finally critical literacy, associated with capabilities required for decision-making within complex contexts [86]. Importantly, for application to primary NCD prevention, this definition is based on a salutogenic framework [87], focusing on wellbeing rather than disease, and recognizing the role of individuals and community in determining the potential of available resources in health-related decision making. This correlates strongly with perspectives of capability development required for critical engagement in open-ended social issues [23].

#### 3.5.3. Scientific Literacy

Scientific literacy is associated with the ability to use scientific knowledge and understanding in decision-making at personal, community and societal levels [88]. Therefore, in conjunction with health literacy, is a critical component of intervention design. Scientific literacy describes capabilities enabling suitably motivated individuals to engage in discourse about science and technology to:

- explain natural and technical phenomena;
- evaluate and design scientific enquiry; and
- interpret data and evidence scientifically [89].

This recognizes that attitudes associated with interest and engagement in the role of science, and use of scientific knowledge within society are components of scientific literacy [90]. It identifies that required capabilities include more than just knowledge; however, that knowledge is important. Furthermore, it highlights the importance of understanding processes by which scientific knowledge is derived and validated [89].

Intervention design should support development of capabilities associated with scientific literacy within three increasingly complex levels of engagement: personal/family, community, and societal (nationally, regionally, and globally). They are moderated by development of attitudes and dispositions promoting engagement in social issues. As such, scientific literacy contributes to the matrix of capabilities required for critical, informed, and responsible citizenship [91] required to enable societal interaction with NCD and DOHaD evidence.

#### 3.5.4. Self Efficacy

The matrix of capabilities described above contributes to individuals or groups engaging with and acting upon scientific and sociological evidence. The extent to which intention leads to sustained action is impacted by self-efficacy and the social/emotional environment. Self-efficacy is the extent to which individuals or communities believe they have the capacity to accomplish a task. Self-efficacy may fluctuate. It is influenced by past experiences, vicarious experiences (modeling from significant others), verbal persuasion, and physiological feedback [92]. Self-efficacy understanding and development should be integrated into intervention design.

### 3.6. Investing in Teachers: Developers and Facilitators of School-Based Interventions

Teacher quality, preparation, beliefs, and commitment are significant controllable factors that enable educational change and influence student learning [93,94]. Health workforce capacity development is an agreed NCD prevention and control strategy [95]. Where the workforce comprises teachers, intervention design must include teacher professional learning and development (PLD) encompassing education, health, and science.

Within multi-sectoral partnerships, lead providers are likely to develop intervention frameworks and core resources, enabling schools to lead intervention adaptation to create contextually appropriate programs contributing towards educational and health goals in each community. Participatory teacher research, a collaborative process whereby teachers, via critical analysis, develop, implement, and evaluate education interventions, is an effective form of PLD to facilitate change [96]. This requires teachers to be learners, a fundamental condition for educational development [97]. This process is inclusive of pedagogy, health and science, and should identify how NCDs/DOHaD can be usefully employed as contexts for learning, contributing towards both health and education goals [98].

Through PLD processes, teachers will adapt programs to ensure they are supportive of learning for and within school communities. This addresses the ever-present challenge of diversity within and between schools, suggested as a reason why evidence-informed health policies and practice applied to school-based health promotion do not always yield similar outcomes [99].

Schools are constantly challenged by change and the need to respond to change [30]. Diversity within and between schools, created by multiple dynamic social, cultural and economic factors, contributes to this complexity. When diversity is not addressed, achievement variance (social and academic) is magnified, indicating under-serving of some groups [100]. Non-prescriptive or devolved education policies (such as national curricula) promote school-level autonomy, creating opportunities to address heterogeneity. This recognizes that because of complexity, application of best practice is frequently not legitimate in school settings. Educators are required to be adaptive experts, “*retrieving, organizing and applying professional knowledge in light of the challenges and needs presented by the students they are teaching”* [29]. This reflects the teaching as an inquiry cycle [97], promoting evidence-based actions by teachers, not as generalizations, but as differentiated responsiveness, ensuring that learning is meaningfully and efficiently directed to all students [93]. Difficulties related to heterogeneity suggest program design may lack adequate educational input, including opportunities at the outset for school-level contextualization followed by development/adaptation in response to reflective critique.

## 4. Conclusions

Biological and sociological evidence points to the potential of schools as a setting for interventions that support transgenerational interruption of the NCD/obesity cycle; however, historically, school-based health interventions have been problematic. Recognition of evidence from multiple sectors and disciplines offers the opportunity to address these historical challenges, which may have occurred due to lack of true partnerships demonstrating mutual respect within multi-sectoral engagement.

On investigation, educators identify in NCD/DOHaD socio-scientific issues of relevance to multiple communities with significant potential as contexts to facilitate learning. When the breadth of later-life health issues impacted by early life environment is linked to social determinants such as poverty, food security, sustainable energy, climate change and technologies, the potential of the context across learning areas ranging from science and health to humanities, arts and languages is identified. This breadth enables multiple exposures via related learning experiences, increasing intervention dose and strength.

Educators need to create the conditions in which adolescents can be empowered as agents of change with respect to obesity, NCDs and socio-ecological impacts. This can only occur when educators are themselves empowered as facilitators of this change, a process that requires transactional engagement between education, health and science communities so that collectively the opportunities can be recognized and enabled.

In effective school-based partnerships, intervention design, while co-constructed, will be led by educators, ensuring links to the core mission of schools. Interventions should:

- support educational and health goals in equal measure;
- be underpinned by appropriate pedagogies utilizing constructs such as student-centered learning, constructivist and inquiry-based epistemologies and epistemic thinking;
- support fulfillment of national-level curriculum, assessment and pastoral care policies and objectives;
- enable integration into school-level curriculum and policies (academic and pastoral), and support strategic development goals;
- provide resources that are designed to be adapted by teachers to meet social, cultural, pastoral and academic needs within their school community and classes, thus supporting development for all learners;
- be evaluated via protocols that are inclusive of the school community and the sectoral partners; and
- acknowledge that learning environments, scientific and health evidence are dynamic constructs, and that programs should evolve over time.

There are currently some programs addressing the potential for DOHaD informed adolescent interventions based on the principles described in this paper occurring in a range of socio-cultural settings. Evidence is emerging, but needs to be developed. The complexity of the school setting, and NCD risk is challenging, and there is room for further exploration of interactions such as those between the school, the adolescent, the family and the community. Research into how such partnerships are developed and maintained should be prioritized, as should publication of evidence that is inclusive of education perspectives. Examination of the impact of program initiation at different phases of child development (for example pre vs. post pubescent), the impact of ongoing program engagement from childhood into adolescence, and the potential for family-involvement should be explored in greater depth. Detailed analysis of intervention tools and empirical evidence relating to education and health, including case studies from a range of contexts and eventually prospective studies, are required.

## Abbreviations

The following abbreviations are used in this manuscript:

DeSeCo	Development and Selection of Competencies
DOHaD	Developmental Origins of Health and Disease
ECHO	Commission on Ending Childhood Obesity
NCDs	Noncommunicable diseases
PLD	Professional Learning and Development
UK	United Kingdom
UN	United Nations
WHO	World Health Organization
